# Pneumonia Caused by Community-Acquired Methicillin-Resistant Staphylococcus aureus Positive for Exfoliative Toxin A and Secondary to Allergic Bronchopulmonary Aspergillosis

**DOI:** 10.7759/cureus.25334

**Published:** 2022-05-25

**Authors:** Junko Itano, Yasushi Tanimoto, Tomoka Nishimura, Kotaro Aoki, Goro Kimura

**Affiliations:** 1 Department of Allergy and Respiratory Medicine, National Hospital Organization Minami-Okayama Medical Center, Okayama, JPN; 2 Department of Microbiology and Infectious Diseases, Toho University School of Medicine, Tokyo, JPN

**Keywords:** exfoliative toxin a, sequence type 121, whole-genome sequencing, community-acquired pneumonia, community-acquired methicillin-resistant staphylococcus aureus

## Abstract

Community-acquired methicillin-resistant *Staphylococcus aureus* (CA-MRSA) causes severe pneumonia. Previous reports found that CA-MRSA producing the Panton-Valentine leukocidin (PVL) or toxic shock syndrome toxin-1 (TSST-1) triggered severe necrotizing pneumonia. However, other toxins and genetic factors responsible for CA-MRSA pneumonia are rarely analyzed in Japan. In this study, we performed whole-genome sequencing (WGS) to analyze the clinical features of CA-MRSA genetically. As a result, we identified a strain with a rare sequence-type of MRSA.

Herein, we present a case of CA-MRSA pneumonia in a 64-year-old woman. Her condition improved rapidly with vancomycin therapy. Draft WGS led to identifying the genotype and virulence factors and showed that the strain was a rare sequence-type of MRSA with the following characteristics: staphylococcal cassette chromosome *mec* (SCC*mec*) type IV, sequence type 121, exfoliative toxin A-positive, and specific staphylococcal protein A type t5110. To the best of our knowledge, a strain with this profile has not been previously reported. Our findings provide new insights into CA-MRSA pneumonia and its genetic and clinical features. Therefore, we recommend accumulating genetic profiles of CA-MRSA pneumonia to identify genetic features and the clinical characteristics of the patients.

## Introduction

*Staphylococcus aureus* is a Gram-positive bacterium that causes severe skin and soft-tissue infections like abscesses, cellulitis, and impetigo. Severe infections caused by *S. aureus* are osteomyelitis and sepsis [1]. The resistance of *S. aureus* to β-lactam antibiotics depends on the mobile genetic composition of the staphylococcal chromosome cassette *mec* (SCC*mec*) [2]. Methicillin-resistant *Staphylococcus aureus* (MRSA) infections have been classified as hospital-acquired and community-acquired (HA-MRSA and CA-MRSA). HA-MRSA infections are associated with life-threatening illnesses and antimicrobial resistance [3] and are distinguished from CA-MRSA based on patient characteristics [4] and genotypic classification [3]. MRSA strains are genetically classified using SCC*mec* typing, multilocus sequence typing (MLST), and staphylococcal protein A (*spa*) typing [5]. Next-generation whole-genome sequencing (WGS) has also been used to study MRSA [6]. Here, we present a case of CA-MRSA pneumonia that developed secondary to allergic bronchopulmonary aspergillosis (ABPA). Genetic analysis of the strain using draft WGS was performed.

## Case presentation

A 64-year-old woman presented to our hospital with a three-week history of fever, cough, and dyspnea. She had been diagnosed with ABPA more than 20 years earlier and was under current treatment with an inhaled corticosteroid and long-acting beta-2-agonist. She had not received any antimicrobial treatment for eight years, except for a three-day course of azithromycin for acute bronchitis four months earlier. Her family history was unremarkable, and there was no history of smoking, pet breeding, overseas travel, or dust exposure.

On physical examination, her temperature was 37.9°C, her heart rate was 82 beats/min, and her percutaneous arterial oxygen saturation was 92% in room air. Upon physical examination, there were no other notable findings except for bilateral coarse crackles in the lower lung. No injury or skin irritation was seen. Her laboratory results included a WBC count of 21,900/mm3 (89.4% neutrophils, 7.1% lymphocytes, 0.1% eosinophils, 0.1% basophils, and 3.3% monocytes) and a C-reactive protein level of 9.12 mg/dL (Table 1). Blood cultures and tests for *Streptococcus pneumoniae* and *Legionella* urinary antigens were negative.

**Table 1:** Laboratory data. Alb: Albumin; ALT: Alanine transaminase; APTT: Activated partial thromboplastin time; AST: Aspartate transaminase; Ba: Basophils; BUN: Blood urea nitrogen; Cl: Chloride; Cre: Creatinine; CRP: C-reactive protein; Eo: Eosinophils; Fib: Fibrinogen; Hb: Hemoglobin; Ht: Hematocrit; K: Potassium; LDH: Lactate dehydrogenase; Ly: Lymphocytes; Mo: Monocytes; Na: Sodium; Neu: Neutrophils; PCT: Procalcitonin; Plt: Platelets; PT: Prothrombin time; γ-GTP: Gamma-glutamyl transpeptidase.

Blood tests	Results		Reference range
WBC	21,900	/mm^3^	3,500-9,100
Neu	89.4	%	35.0-71.0
Ly	7.1	%	20.0-53.0
Mo	3.3	%	2.0-12.0
Eo	0.1	%	0.0-8.0
Ba	0.1	%	0.0-2.0
RBC	454	×10^4^/mm^3^	370-500
Hb	15	g/dL	11.0-15.0
Ht	44.5	%	33.0-45.0
Plt	603	×10^3^/mm^3^	130-373
CRP	9.12	mg/dL	0.00-0.30
PCT	0.02	ng/dL	0.00-0.50
Alb	3.6	mg/dL	4.0-5.0
AST	15	U/L	13-33
ALT	11	U/L	6.0-27.0
LDH	179	U/L	119-229
γGTP	19	U/L	10-47
Na	144	mEq/L	138-146
K	4.4	mEq/L	3.6-4.9
Cl	102	mEq/L	99-109
BUN	13	mg/dL	8.0-22.0
Cre	0.61	mg/dL	0.40-0.70
PT	12.6	sec	10.0-14.0
APTT	25.7	sec	24.0-40.0
Fib	604.4	mg/dL	150.0-400.0
D-dimer	1.1	µg/mL	0.0-1.0

**Figure 1:**
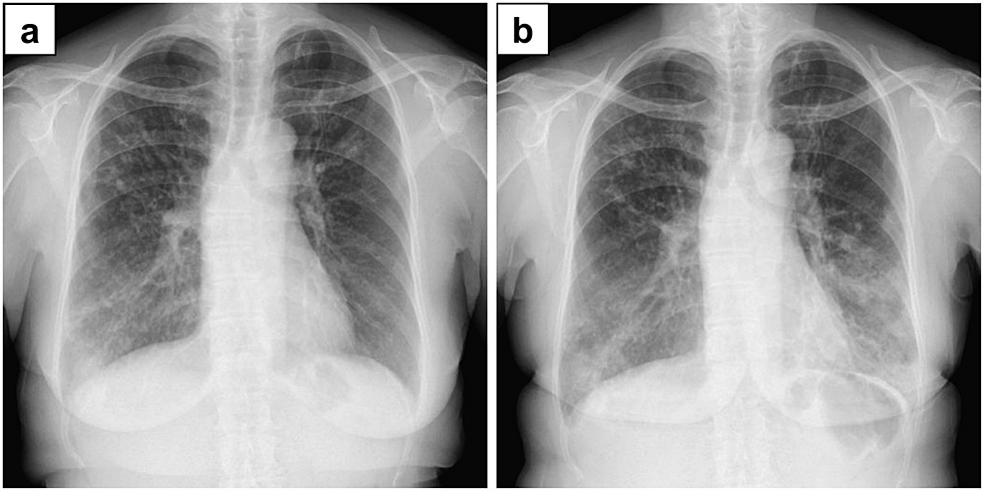
Chest X-ray. Chest X-ray films obtained six months before admission (a) and on admission to our hospital (b). Chest radiograph showed consolidations in the bilateral lower lung fields.

**Figure 2:**
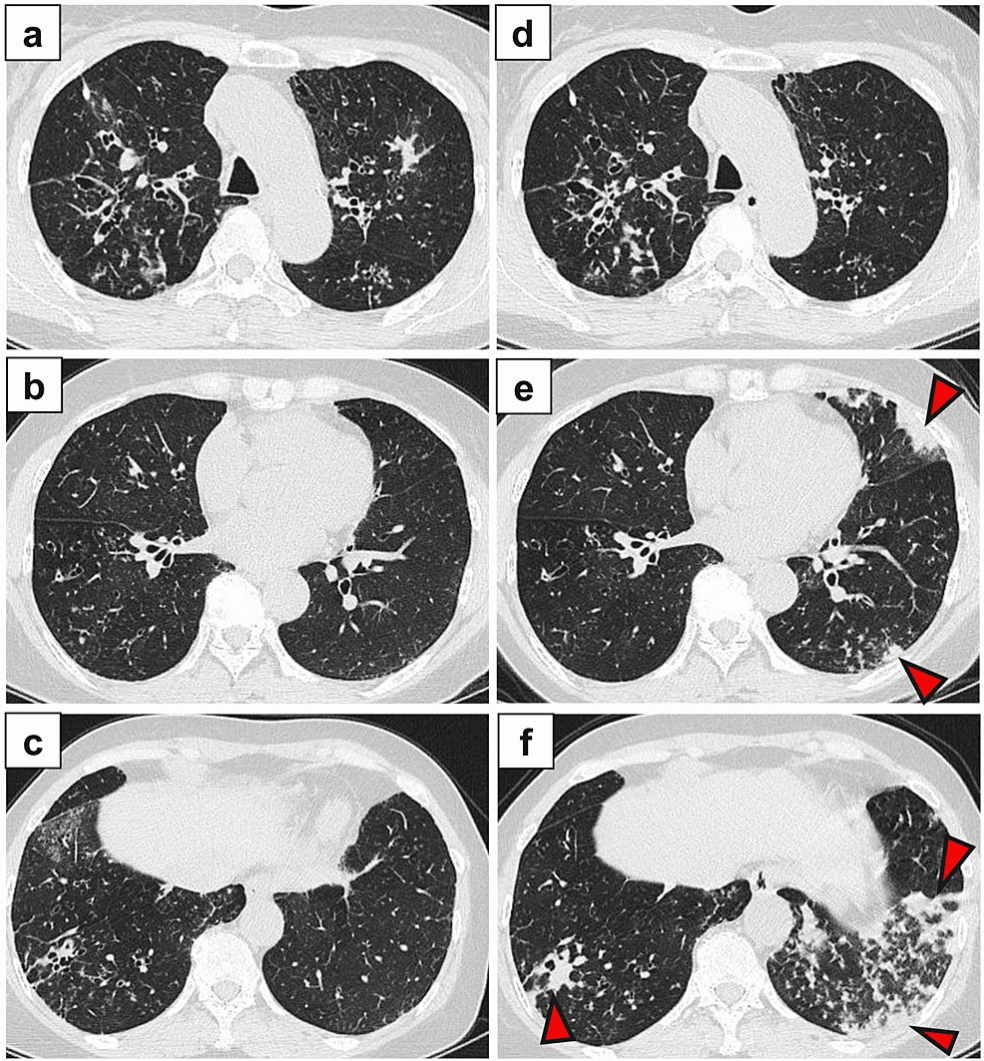
Chest computed tomography. (a-c) Chest CT revealed bronchiectasis with bronchial wall thickening and mucoid impaction in the bilateral lung fields. (d-f) After six months, CT revealed bronchial wall thickening and consolidations (red arrowheads) in the left upper lobe and bilateral lower lobes.

Chest radiographs were obtained six months before and at the time of presentation. In the former, bronchial wall thickening and small nodules were noted (Figure 1a). CT performed at that time showed bronchiectasis with bronchial wall thickening and mucoid impaction in the bilateral lung fields (Figure 2a-2c). Together, these observations suggested ABPA. Images obtained at the time of presentation were consistent with symptoms of fever and cough. Chest radiographs showed consolidations in the bilateral lower lung fields (Figure 1b), and CT revealed bronchial wall thickening and consolidations in the left upper lobe and bilateral lower lobes (Figure 2d-2f).

Based on these results, the patient was diagnosed with community-acquired pneumonia secondary to ABPA. Sputum smear examination that included Gram staining revealed Gram-positive cocci and phagocytic WBCs. Treatment at the outpatient clinic comprised IV administration of ceftriaxone (2 g/day) and oral administration of levofloxacin (500 mg/day). However, as the patient’s cough and dyspnea persisted, she was admitted to our hospital one day later, where she was started on IV sulbactam/ampicillin (SBT/ABPC; 3 g thrice daily).

A sputum specimen collected at the outpatient clinic was washed with normal saline and inoculated onto trypticase soy agar with 5% sheep blood agar (TSA II; Becton Dickinson, Tokyo, Japan). All specimens were cultured overnight at 35°C in a humidified incubator with 5% carbon dioxide. The culture was analyzed using an automated matrix-assisted laser desorption-ionization time of flight mass spectrometry microbial identification system (VITEK MSTM, SYSMEX bioMérieux Co., Ltd., Tokyo, Japan) following the manufacturer’s protocol. The strain was identified as *S. aureus* using the SARAMIS database. Antimicrobial susceptibility testing of the isolated bacterium was performed using the VITEK2 (BioMérieux Co., Ltd., Tokyo, Japan) automated system, which showed that the strain was MRSA, resistant to oxacillin and several other antimicrobial agents but susceptible to vancomycin.

**Figure 3:**
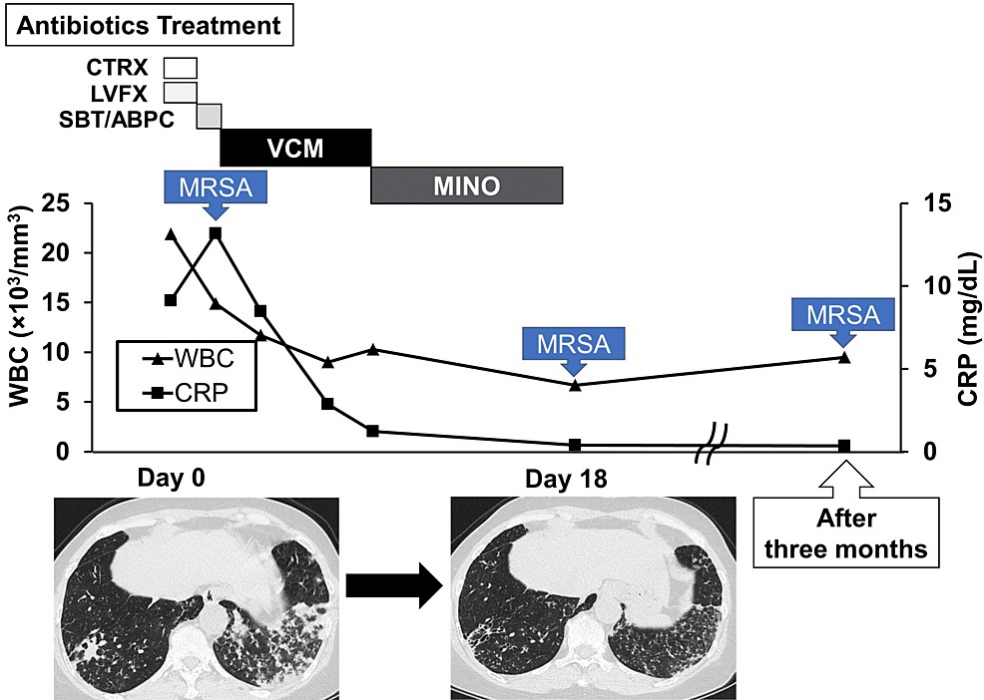
Treatment and clinical course of the patient. The patient received IV administration of ceftriaxone (2 g/day) and oral administration of levofloxacin (500 mg/day) in the outpatient clinic. She was hospitalized the following day and received an IV administration of sulbactam/ampicillin (3 g, three times for one day). In addition, IV vancomycin (0.5 g, twice daily) was administered for five days, with subsequent minocycline (200 mg/day) treatment for eight days. The WBC count and C-reactive protein concentrations over time are shown. Chest CT findings before admission (day 0) and after treatment (day 18) revealed an improvement in the consolidations of the bilateral lower lobes. CRP: C-reactive protein; CTRX: Ceftriaxone; LVFX: Levofloxacin; MINO: Minocycline; MRSA: Methicillin-resistant *Staphylococcus aureus*;* *SBT/ABPC: Sulbactam/Ampicillin; VCM: Vancomycin.

Treatment was therefore switched from SBT/ABPC to IV vancomycin (0.5 g twice daily) for MRSA. After five days, the patient’s symptoms rapidly improved. Vancomycin was then replaced by minocycline (200 mg/day), and the patient was discharged. A CT scan performed 18 days after the first diagnosis showed improvement in the consolidations of the bilateral lower lobes (Figure 3). Subsequently, the patient was monitored carefully at the outpatient clinic. However, despite the disappearance of her symptoms, three months and 18 days after she had been placed on antimicrobial therapy, MRSA was again detected in the patient’s sputum, revealing antimicrobial resistance.

To further investigate the pathogenesis of the infection, the strain was isolated from sputum and analyzed at the Department of Microbiology and Infectious Diseases of Toho University, Japan. A drug susceptibility test was also conducted at Toho University to confirm the drug resistance of the previously stored strain isolated from the sputum. In addition, minimum inhibitory concentration (MIC) testing for antimicrobial agents was repeated using a Dry Plate Eiken DP32 (Eiken, Tokyo, Japan). As shown in Table 2, the strain was resistant to oxacillin (MPIPC) (MIC: >4 mg/L) and cefoxitin (MIC: ≥8 mg/L), and was confirmed to be MRSA according to Clinical and Laboratory Standards Institute guidelines (2021) [7]. 

**Table 2:** Susceptibilities for antimicrobial agents. ABPC: Ampicillin; CEZ: Cefazolin; CFX: Cefoxitin; CLDM: Clindamycin; CMZ: Cefmetazole; EM: Erythromycin; GM: Gentamicin; IPM: Imipenem; LVFX: Levofloxacin; LZD: Linezolid; MIC: Minimum inhibitory concentration; MINO: Minocycline; MPIPC: Oxacillin; R: Resistant; S: Susceptible; ST: Sulfamethoxazole-trimethoprim; TEIC: Teicoplanin; VCM: Vancomycin.

Antimicrobial agents	MIC (mg/L)	Interpretation
MPIPC	>4	R
CFX	8	R
ABPC	16	R
CEZ	2	R
CMZ	4	R
IPM	≤0.25	R
GM	>8	R
MINO	≤2	S
EM	>4	R
CLDM	0.12	S
VCM	1	S
TEIC	≤0.5	S
LZD	2	S
ST	≤9.5/0.5	S
LVFX	1	S

The isolate’s genomic DNA (TUM20400) was extracted and analyzed by draft WGS analysis using the MiSeq platform system (Illumina, San Diego, CA, USA) and the MiSeq reagent kit v3,600 cycle (Illumina, 350 bp/250 bp paired-end reads). Draft genome sequences (contigs) were generated from the MiSeq data using SPAdes version 3.15.2 [8]. The contigs were analyzed by MLST, SCC*mec* typing, and *spa* typing; a virulence search was performed using MLST 2.0, SCC*mec*Finder 1.2, *spa*Typer 1.0, and Virulence Finder 2.0, respectively, using web tools from the Center for Genomic Epidemiology (http://www.genomicepidemiology.org/). In addition, the presence of the exfoliative toxin (ET)-encoding genes *eta*, *etb*, and *etd* was searched using an in silico polymerase chain reaction [9]. The genotypes and virulence factors are shown in Table 3. Draft WGS revealed that the strain was *eta*-positive, SCC*mec* type IV, sequence type (ST) 121, and *spa* type t5110.

**Table 3:** Genotype and virulence factors. MLST: Multilocus sequence typing; SCC*mec:* Staphylococcal cassette chromosome *mec*; *spa:* Staphylococcal protein A; ST: Sequence type.

Isolate ID	SCC*mec*	MLST	spa	Virulence genes
TUM 20400	IV	ST121	t5110	*aur*,* splA*,* splB*, *scn*, *sak*, *hlgA*,* hlgB*,*hlgC*, *lukD*,* lukE*, *eta*, *seg*, *sei*,* sem*,* sen*, *seu*.

According to the drug susceptibility test, draft WGS analysis, and criteria proposed by Naimi TS et al. [4], the patient was diagnosed with pneumonia caused by CA-MRSA secondary to ABPA. She rapidly improved and is currently monitored as an outpatient. One year after treatment, there has not been any recurrence of pneumonia. The draft WGS data of TUM20400 have been deposited in the DNA Data Bank of Japan under accession number BQKF01000001-BQKF01000068.

## Discussion

This case report described a patient who developed CA-MRSA pneumonia secondary to ABPA. To shed light on the clinical course, toxin, and genotypes of the strains responsible for CA-MRSA in Japan, we reviewed previous reports that included chest radiographs, CT findings, and data on toxin production and genetic analysis using SCC*mec* typing, MLST, and *spa* typing. Our search led to reports of only four other adult patients for whom the above information was provided [10-12]. Panton-Valentine leukocidin (PVL) and toxic shock syndrome toxin-1 (TSST-1) have been shown to cause severe pneumonia [10,11]. The strains resulting in the deaths of three patients were: ST22/*spa*-t005/SCC*mec* IVa producing both PVL and TSST-1 [10], ST5/*spa*-t002/SCC*mec* II producing TSST-1 [11], and ST121/*spa*-t5110/SCC*mec* V, without producing PVL, TSST-1 and ETA [12]. In our patient, the ETA-producing clone was identified as ST121/*spa*-t5110/SCC*mec* IV. This MRSA isolate has not been previously reported.

In Japan, the ST8/SCC*mec* IVl (ST8 CA-MRSA/J) clone comprises 16.2-34.4% of CA-MRSA genomes. Strains belonging to ST8 CA-MRSA/J have been shown to cause infectious diseases [13]. Strains belonging to ST121 have been isolated in Africa, Asia, and Europe, with 90% of them reported to be methicillin-susceptible *S. aureus*; the others contained the PVL-encoding gene and showed high virulence [14]. Although CA-MRSA pneumonia caused by a strain belonging to ST121 and not producing PVL, TSST-1, or ETA was reported, the clinical background of the case was different than that of ours, as the patient in the former was 92 years old, had a history of cerebral infarction, and died of aspiration pneumonia [12].

CA-MRSA pneumonia is characterized by rapid tissue destruction, cavity formation, and necrotizing pneumonia [10,11,15]. However, our patient had neither cavity formation nor necrotizing pneumonia; her CT findings revealed only consolidation. This case, therefore, differed from previously reported cases of CA-MRSA, which included necrotizing pneumonia [10,11,15]. In the two cases reported by Hayakawa K et al. [10], the strains were positive for PVL and TSST-1. One of the patients died, whereas our patient rapidly improved after the initiation of vancomycin therapy. Moreover, to our knowledge, prior to our case, there have been no other cases of ETA-producing strains causing CA-MRSA pneumonia in adults.

ET produced by *S. aureus* causes staphylococcal scaling skin syndrome (SSSS), generalized exfoliative syndrome, and bullous impetigo. Among the 283 strains causing SSSS, 36% were shown to carry *eta*, and 54% were shown to carry both *eta* and *etb*; 75% belonged to ST121 [16]. These data suggest that strains of *S. aureus* producing ET toxin and belonging to ST121 cause skin lesions. While the relationship between ETA and CA-MRSA pneumonia remains unclear, our data indicate that our patient was infected with a rare subclone of MRSA belonging to ST121 and producing ETA.

ABPA is a chronic inflammatory airway disease caused by an allergic reaction to fungal species. Chest images of patients with ABPA show mucus plugs and central bronchiectasis [17]. ABPA is typically treated with corticosteroids, whose long-term use in this setting is associated with secondary infections, such as by nontuberculous mycobacteria and *Pseudomonas aeruginosa* [18]. By contrast, we are aware of only one report of MRSA pneumonia secondary to ABPA. In that case, the patient developed bilateral septic arthritis of the knees, but the genetic characteristics of the MRSA were not determined [19].

MRSA becomes established in the lower respiratory tract in patients with chronic lung diseases, such as chronic obstructive pulmonary disease. A disruption of the balance between host defenses and bacterial virulence can lead to infections, such as those resulting in pneumonia [20]. Our patient had ABPA for more than 20 years, and MRSA might have been present in the airway epithelium. While CA-MRSA pneumonia often becomes severe, our patient improved rapidly after a short period of vancomycin administration.

## Conclusions

In summary, we reported a case of CA-MRSA pneumonia caused by a rare MRSA subclone in a patient with ABPA. As there have been few reports of MRSA-induced pneumonia associated with ABPA, the genetic and clinical features of the responsible bacterial strains are poorly understood. Therefore, we recommend accumulating genetic profiles of CA-MRSA pneumonia to identify genetic features and the clinical characteristics of the patients.
